# The impact of COVID-19 prevention and control policy adjustment on anxiety, depression and coping behavior in China: a cross-sectional online survey, 21–28 December, 2022

**DOI:** 10.1186/s12889-023-16699-0

**Published:** 2023-09-15

**Authors:** Mingyu Gu, Tingting Qin, Kun Qiao, Xinyuan Bai, Yao Wang, Yutong Yang, Yu Bai, Jie Gao, Xingming Li

**Affiliations:** School of Public Health, Capital Medical University, Beijing, 100069 China

**Keywords:** COVID-19, Policy adjustment, Anxiety, Depression, Coping behavior, Cross-sectional study

## Abstract

**Background:**

Following external situation reports, individuals perceive risks, experience different emotional reactions, and further change their behaviors. Therefor people’s psychology will also be affected by adjustment of COVID-19 epidemic prevention and control policy, but it remains unknown what kind of coping behaviors will be produced due to psychology. This study defines coping behavior as “medical behavior and irrational consumption behavior after the adjustment of COVID-19 epidemic prevention and control policy in China”, assesses the prevalence of negative emotions in the Chinese population after policy adjustments, and explores how negative emotions affect people’s coping behaviors, conducts baseline research, provides references and suggestions for policy formulation.

**Methods:**

A cross-sectional online survey was conducted during 21–28 December 2022, included sociodemographic characteristics, COVID-19 infection and irrational purchase behavior, psychological assessment, and opinion polling. Depression and anxiety status are assessed by PHQ-9 and GAD-7. The relationship between anxiety, depression and coping behavior was analyzed by Pearson χ^2^ test, Fisher’s exact test and logistic regression.

**Results:**

A total of 3995 infected participants were included in this study, of which 2363(59.1%) and 1194(29.9%) had depression and anxiety. There was a significant difference in clinical treatment and irrational purchase behavior between different level of depression and anxiety. Depression was a risk factor for self- medication (OR = 1.254), seeking professional treatment (OR = 1.215), using online services of medical institutions (OR = 1.320), large-scale purchases of medicines (OR = 1.154) and masks (OR = 1.096). Anxiety was a risk factor for seeking professional treatment (OR = 1.285) and large-scale purchases of masks (OR = 1.168).

**Conclusion:**

After the adjustment of COVID-19 epidemic prevention and control policy, patient risk perception can increase depression and anxiety. We found that associated with depression, COVID-19 patients are more likely to have medical behaviors such as self- medication, seeking professional treatment, using online services of medical institutions, and storage behaviors of medicines and masks; and anxiety associated with the coping behavior of patients to seek professional treatment and store masks in large quantities. We should improve people’s mental health, and on the other hand, we should give people effective psychological education during the epidemic. Therefore, we should set up psychological outpatient clinics in community health institutions, expanding mental health screening and guidance; relying on the psychological outpatient clinic, establish groups of people with depression or anxiety to carry out COVID-19 health education and peer education, to reduce adverse drug reactions, avoid panic seeking professional treatment and irrational purchase behavior, and protect public mental health.

**Trial registration:**

This study has been approved by the Medical Ethics Committee of Capital Medical University (2023SY086), and informed consent was obtained from the study subjects before the investigation.

## Background

 Since the beginning of the COVID-19 pandemic, China implemented the Zero-COVID policy and has promptly blocked hundreds of COVID-19 outbreaks that are associated with imported cases. The Zero-COVID policy seeks to completely stop community transmission by very stringent interventions like lockdowns, mobility restrictions, mass testing, contact tracing, case isolation, close contact quarantine, and border quarantine [1, 2]. However, with the shortened incubation period, stronger transmission ability, faster transmission speed, weakened pathogenicity, and stronger immune escape ability, Omicron has become a pandemic in China and the world. It brings new challenges on the healthcare system. Facing the new situation of the COVID-19 epidemic, China has recently started to adjust its response strategy to COVID-19 after two years of implementing the Zero-COVID policy. On November 11, 2022, National Health Commission of the People’s Republic of China released " Notice on Further Optimizing the Prevention and Control Measures of COVID-19 response” (referred to as “20 Measures”) [3], and further implemented " Notice on Further Optimizing and Implementing the Prevention and Control Measures of COVID-19 Epidemic " (referred to as “10 Measures”) [4] on December 7. The 20 measures include reducing isolation periods, stopping identify Secondary close contacts, adjusting the categories of COVID-19 risk areas, stopping of mass testing, relaxing travel restrictions and associated testing, boosting healthcare resources and stockpiling medicines, stepping up the coverage of booster vaccination, and improving the protection of key priority groups. Based on the 20 measures, the 10 measures further narrow the testing range, divide risk areas accurately, reduce isolation periods, and adjust the isolation method.

**Fig. 1:**
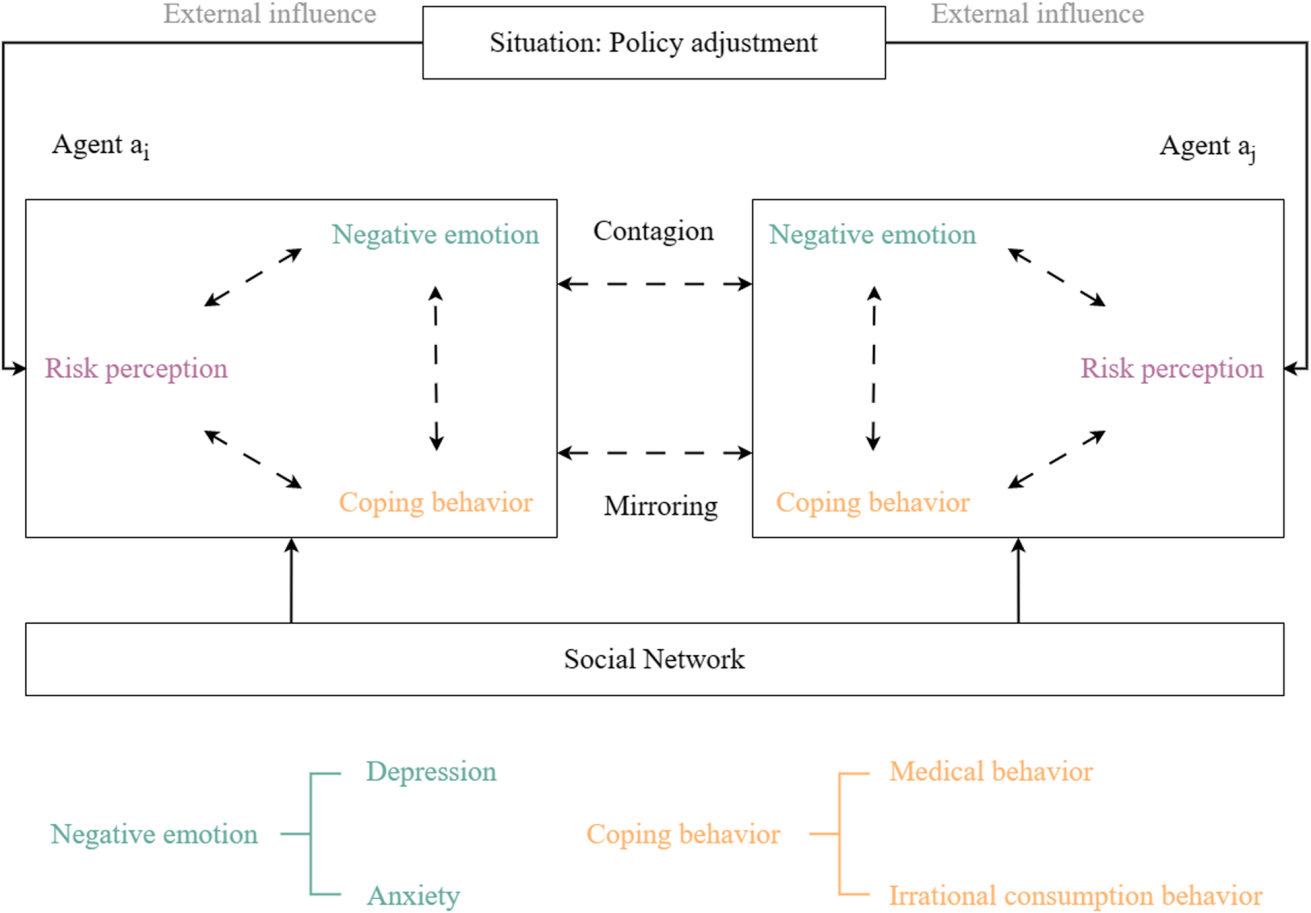
Structure of the individual-based model with influences from the external situation and individual-based social networks

With the release of the “20 Measures” and “10 Measures”, the prevention and control of the COVID-19 epidemic in China has entered a new stage. It can be predicted that when people adjust from the familiar Zero-COVID policy to the new policy, combined with combined with the Omicron pandemic, people’s psychology will also be affected by the change. Zhanwei Du et al. [5] showed that following external situation reports, individuals perceive risks, experience different emotional reactions, and further change their behaviors — usually by following the strengthening process (i.e., risk perception drives emotional reactions, and emotional reactions affect behaviors, Fig. 1). Inversely, resulting behaviors should reduce individuals’ emotions (e.g., anxiety and stress) and risk perception. However, after the policy adjustment, what kind of psychological changes have taken place in people, and what coping behaviors have been produced due to the psychological changes are still unknown. Therefore, this study focuses on assessing the prevalence of negative emotions in the Chinese population after policy adjustments, and explores how negative emotions affect people’s coping behaviors, conducts baseline research, and provides references and suggestions for policy formulation.

## Materials and methods

### Study design and participants

This study conducted a cross-sectional survey in China after the adjustment of COVID-19 epidemic prevention and control policy and recruited participants using network interception survey and convenience sampling. On December 21, 2022, the online survey questionnaire was designed on the basis of the Chinese online questionnaire survey platform: Wen Juan Xing (Changsha Ranxing Information Technology Co., Ltd., Hunan, China), and the questionnaire was distributed through the online social platform: WeChat (Tencent Ltd., Shenzhen, China), a Chinese social media APP. Users can voluntarily participate in the survey by scanning the QR code and were encouraged to forward it to relatives, friends, colleagues, etc. By December 28, 2022, a total of 5,310 questionnaires have been collected, involving 34 provinces in China and overseas regions.

**Fig. 2:**
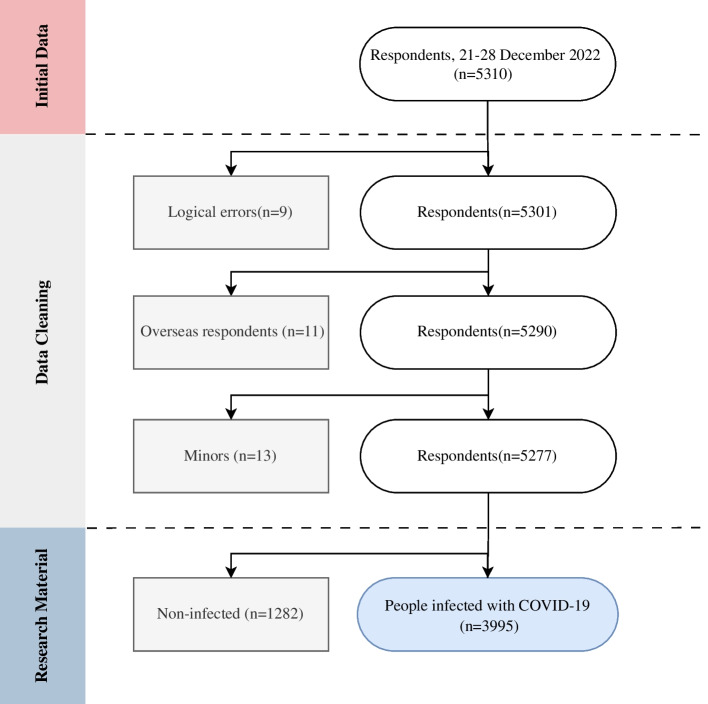
Flowchart of the study design

Inclusion criteria: (1) Willing to participate in this study; (2) Smartphone users or filled by family members; (3) Knowing if he/she was infected and having a certain understanding of COVID-19. Exclusion criteria: (1) Incomplete questionnaire; (2) Logical error; (3) Overseas users; (4) Minors. According to the inclusion and exclusion criteria, 33 data were excluded, among which, 9 data had logical errors, 11 users live in overseas and 13 minors. Collected a total of 5277 valid data and the effective rate of questionnaire recovery was 99.4%. Finally, included 3995 data who reported infection with COVID-19 in this study (Fig. 2).

Collected participant informed consent before the investigation.

Infection rate of the fifth wave of COVID-19 in Hong Kong is 14.5% [6]. $\delta =\pm 2\%$,$\alpha =0.05$, ${\mu }_{\alpha }=1.96$. Estimate the number of samples, the results showed that survey at least 1190 participants as sample size. The calculation formula and process are as formula 1:

1 $$\begin{array}{c}n=\frac{\mu_\alpha^2\;p\left(1-p\right)}{\delta^2}=\frac{{1.96}^2\;\times\;0.145\;\times\;0.855}{{0.02}^2}\end{array}\approx\;1190$$

**Fig. 3:**
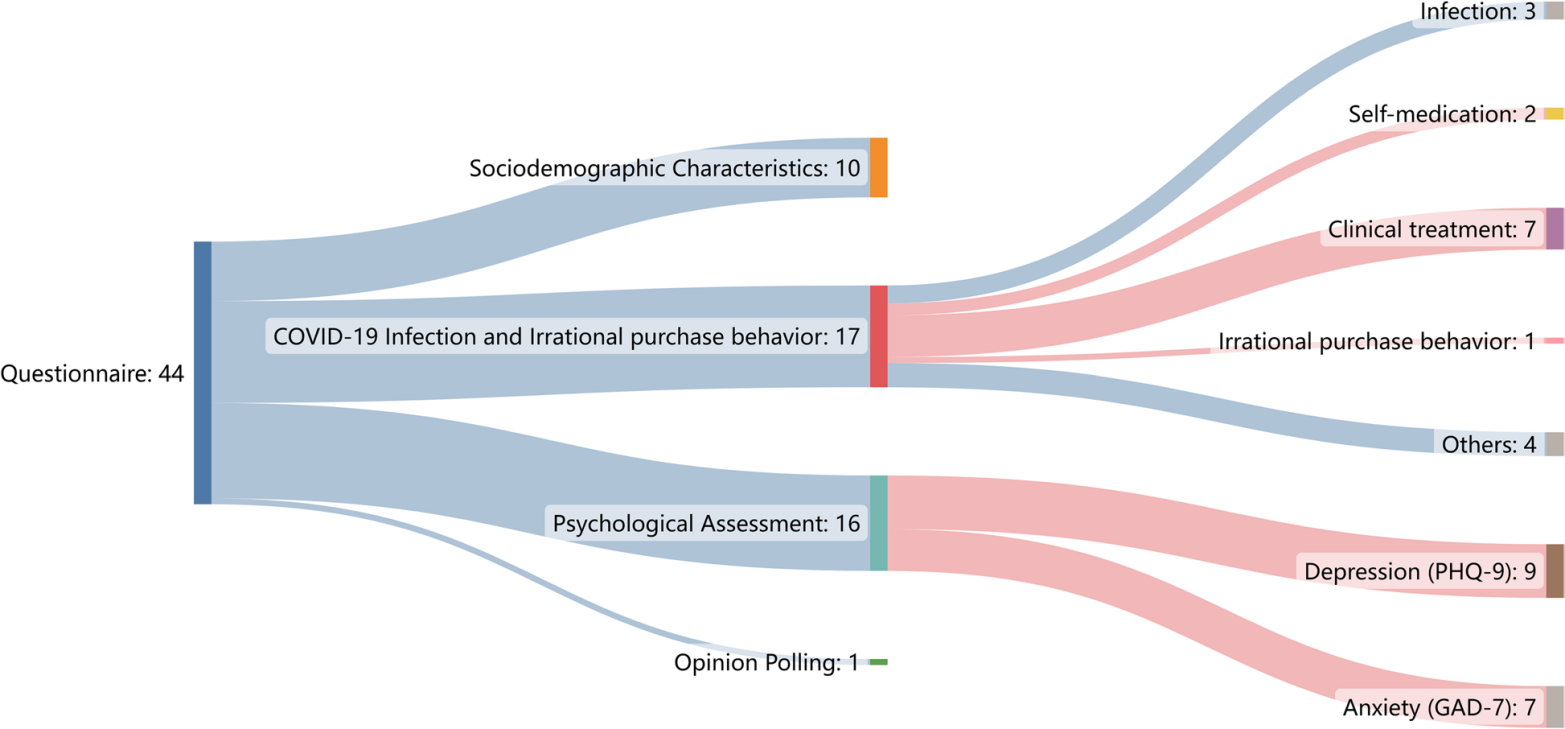
Questionnaire design and structure. The red part is the survey items included in this study; (n) represents the number of survey items

The questionnaire consists of self-designed questionnaires and standard psychological scales, which contain four sections: sociodemographic characteristics, COVID-19 infection and irrational purchase behavior, psychological assessment, and opinion polling (Fig. 3). (1) Socio-demographic characteristics include gender, age, education level, marital status, employment status, monthly personal income, residence area, history of physical illness, and vaccination; (2) COVID-19 infection and irrational purchase behavior include infection, self- medication, clinical treatment, irrational purchase behavior and others; (3) Psychological assessment including assessment of depression and anxiety; (4) Opinion polling.

In order to assess the depression level of the participants, this study used the Patient Health Questionnaire (PHQ-9) scale [7] to measure the depression score. PHQ-9 presented respondents with 9 statements, each with a four-point scale and the total score ranged from 0 to 27. According to the score of 9 statements, the depression is divided into 5 levels: none (0–4), mild (5–9), moderate (10–14), moderately severe (15–19), severe (20–27). Anxiety scores were measured by the General Anxiety Disorder (GAD-7) scale [8]. Like PHQ-9, GAD-7 also presented respondents with 7 statements, each with a four-point scale and the total score ranged from 0 to 21. The anxiety is divided into 4 levels: none (0–4), mild (5–9), moderate (10–14), severe (15–21). The Chinese versions of PHQ-9 and GAD-7 have been demonstrated to have good reliability and validity [9, 10]. In this study, we found that the Cronbach’s alpha of self-designed questionnaire, PHQ-9 and GAD-7 were 0.738, 0.896 and 0.945, respectively, which indicated that the questionnaires had great internal validity.

**Fig. 4:**
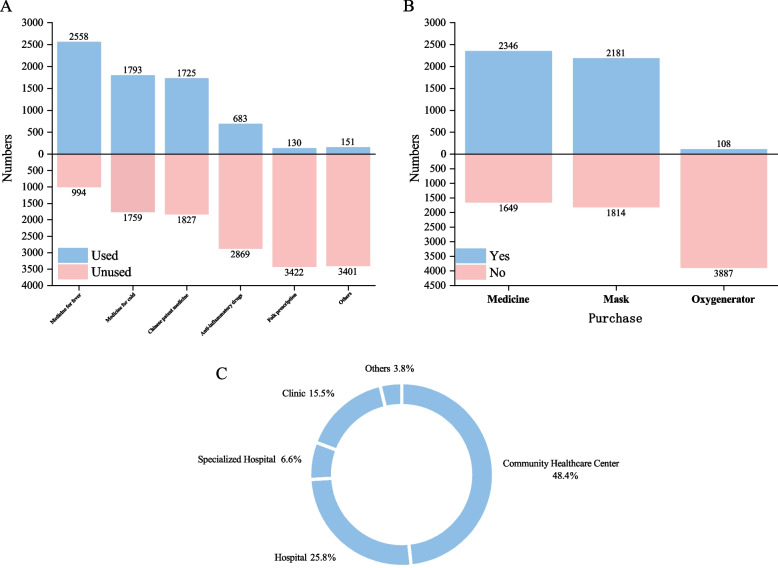
Participants’ self-medication (**A**), clinical treatment (**B**), and irrational purchase behavior (**C**)

Coping behavior is defined as the behaviors that individuals exhibit aimed at responding to environmental challenges [11]. In this study, we define coping behavior as “medical behavior and irrational consumption behavior after the adjustment of COVID-19 epidemic prevention and control policy in China”. In medical behavior, we investigated self-medication and clinical treatment. The World Health Organization (WHO) defines self-medication (SM) as “Self-medication refers to the selection and utilization of medicines to treat self-recognized symptoms or ailments without consulting a physician” [12], Fig. 4A shows the self-medication of the participants. Irrational purchase behavior is narrowly defined as irrational purchase decisions consumers have made when affect by a variety of factors, generally performance are consumers don’t pursuit the utility maximization when they purchase, or don’t consider the income constraint when purchase, or lack of knowledge to judge consumer goods, and so on [13]. In this study, we define irrational purchase behavior as “purchase behavior after the adjustment of COVID-19 epidemic prevention and control policy in China, such as large-scale purchases of medicines, masks, and unreasonable purchases of oxygen generators”, Fig. 4B shows the irrational consumption behavior of the participants. In this study, Clinical treatment is defined as seeking professional treatment in various medical institutions or using online services of medical institutions (such as online consultation, telemedicine, etc.) after being infected with COVID-19, The types of medical institutions visited by the participants are shown in Fig. 4C.

### Statistical analysis

Categorical variables were reported as n (%) (constituent ratio) and continuous variables were summarized as mean ± standard deviation (SD). Pearson χ^2^ test or Fisher’s exact test were used to compare participants’ anxiety as well as depression with medical behaviors and irrational purchase behaviors. The relationships between anxiety as well as depression symptoms and explanatory variables were assessed with binary logistic regression. Reported odds ratios (OR) and 95% confidence intervals (95% CI) of independent variable. All statistical analyses were performed using SPSS statistical software version 28.0. *P*-values < 0.05 were considered statistically significant.

## Results

### Sociodemographic characteristics

A total of 3995 data were included in this study, and all participants were COVID-19 infected. Among all participants, there were 1143 males (28.6%) and 2852 females (71.4%); the age groups were mainly 31–45 years old and 46–60 years old, accounting for 41.3% and 28.7% respectively; most of the participants had associate/Bachelor degree and above (85.6%), more than half of the participants are married, 2947 people (73.8%); in terms of employment status, 2758 people (69.0%) are employed, and the average monthly income is 5000 ~ 10000RMB, accounting for the majority, a total of 1076 people (26.9%); 3687 people (92.3%) live in cities; 3325 people (83.2%) had no history of chronic diseases, and 3421 people (85.6%) had completed 3 doses of vaccination. As shown in Table 1.

**Table 1:** Socio-demographic characteristics of respondents after the adjustment of China’s COVID-19 prevention and control policy in 2023, (*n* = 3995)

Sociodemographic Characteristics	*n*	*%*
Gender		
Male	1143	28.6
Female	2852	71.4
Age(years)		
17 ~ 30	903	22.7
31 ~ 45	1648	41.3
46 ~ 60	1147	28.7
≥61	297	7.4
Education level		
High School diploma or below	574	14.4
Associate/bachelor degree	2199	55.0
Graduate degree	1222	30.6
Marital status		
Unmarried	869	21.8
Married	2947	73.8
Others	179	4.4
Employment status		
Employed	2758	69.0
Retired	505	12.6
Students	487	12.2
Others	245	6.2
Monthly personal income (Yuan)		
<3000	798	20.0
3000 ~ 5000	697	17.4
5000 ~ 1000	1076	26.9
10,000 ~ 15,000	797	19.9
>15,000	627	15.7
Residence area		
Urban	3687	92.3
Rural	308	77
Region		
East	3078	77.0
Central	326	8.2
Western	591	14.8
History of chronic diseases		
Yes	670	16.8
No	3325	83.2
Vaccination		
0	167	4.2
1	32	0.8
2	375	9.4
3	3245	81.2
4	176	4.4

The eastern region includes Beijing, Tianjin, Hebei, Liaoning, Shanghai, Jiangsu, Zhejiang, Fujian, Shandong, Guangdong, and Hainan; the central region includes Shanxi, Jilin, Heilongjiang, Anhui, Jiangxi, Henan, Hubei, and Hunan; the western region Regions include Inner Mongolia, Chongqing, Guangxi, Sichuan, Guizhou, Yunnan, Tibet, Shaanxi, Gansu, Qinghai, Ningxia, and Xinjiang

### Status of medical treatment among participants

According to the results of the Pearson χ^2^ test, we found that there was a significant difference in self- medication, seeking professional treatment, using online services of medical institutions between different level of depression and anxiety, after the adjustment of COVID-19 epidemic prevention and control policy. The occurrence of depression or anxiety affects the proportion of self- medication, seeking professional treatment, using online services of medical institutions (Tables 2 and 3). after the adjustment of COVID-19 epidemic prevention and control policy, people’s medical behavior is affected by depression and anxiety.

### Status of irrational purchase behavior among participants

In addition to purchasing oxygen machines, there was a significant difference in large-scale purchase of medicines and masks between different level of depression and anxiety. Same as medical treatment, the occurrence of depression or anxiety affects the proportion of large-scale purchase of medicines and masks (Tables 4 and 5). After the adjustment of COVID-19 epidemic prevention and control policy, people’s different levels of depression or anxiety are the key factors affecting irrational purchase behavior.

**Table 2:** Chi-square test of depression on medical treatment, n (%)

Medical treatment	None	Mild	Moderate	Moderately severe	Severe	*n*	χ^2^	*P*
Self- medication								
Yes	1406 (86.2)	1370 (90.3)	450 (91.3)	218 (94.8)	108 (88.5)	3552	26.225	<0.001
No	226 (13.8)	148 (9.7)	43 (8.7)	12 (5.2)	14 (11.5)	443		
Seeking professional treatment								
Yes	62 (3.8)	71 (4.7)	47 (9.5)	20 (8.7)	13 (10.7)	213	38.136	<0.001
No	1570 (96.2)	1447 (95.3)	446 (90.5)	210 (91.3)	109 (89.3)	3782		
Using online services of medical institutions								
Yes	53 (3.2)	69 (4.5)	24 (4.9)	22 (9.6)	9 (7.4)	177	22.487	<0.001
No	1579 (96.8)	1449 (95.5)	469 (95.1)	208 (90.4)	113 (92.6)	3818		

**Table 3:** Chi-square test or Fisher’s exact test of anxiety on medical treatment, n (%)

Medical treatment	None	Mild	Moderate	Severe	*n*	χ^2^	*P*
Self- medication							
Yes	2463 (87.9)	806 (91.0)	199 (93.0)	84 (89.4)	3552	10.162	0.017
No	338 (12.1)	80 (9.0)	15 (7.0)	10 (10.6)	443		
Seeking professional treatment							
Yes	117 (4.2)	62 (7.0)	22 (10.3)	12 (12.8)	213	32.946	<0.001
No	2684 (95.8)	824 (93.0)	192 (89.7)	82 (87.2)	3782		
Using online services of medical institutions							
Yes	101 (3.6)	53 (6.0)	19 (8.9)	4 (4.3)	177	19.541^a^	<0.001^a^
No	2700 (96.4)	833 (94.0)	195 (91.1)	90 (95.7)	3818		

a Fisher’s exact test

**Table 4:** Chi-square test or Fisher’s exact test of depression on irrational purchase behavior, n (%)

Irrational purchase behavior	None	Mild	Moderate	Moderately severe	Severe	*n*	χ^2^	*P*
Large-scale purchases of medicines								
Yes	865 (53.0)	946 (62.3)	318 (64.5%)	148 (64.3)	69 (56.6)	2346	40.164	<0.001
No	767 (47.0)	572 (37.7)	175 (35.5)	82 (35.7)	53 (43.4)	1649		
Large-scale purchases of masks								
Yes	808 (49.5)	866 (57.0)	291 (59.0)	136 (59.1)	80 (65.6)	2181	32.458	<0.001
No	824 (50.5)	652 (43.0)	202 (41.0)	94 (40.9)	42 (34.4)	1814		
Unreasonable purchases of oxygen generators								
Yes	34 (2.1)	49 (3.2)	11 (2.2)	8 (3.5)	6 (4.9)	108	7.191^a^	0.104^a^
No	1598 (97.9)	1469 (96.8)	482 (97.8)	222 (96.5)	116 (95.1)	3887		

^a^Fisher’s exact test

**Table 5:** Chi-square test or Fisher’s exact test of anxiety on irrational purchase behavior, n (%)

Irrational purchase behavior	None	Mild	Moderate	Severe	*n*	χ^2^	*P*
Large-scale purchases of medicines							
Yes	1560 (55.7)	594 (67.0)	141 (65.9)	51 (54.3)	2346	41.208	<0.001
No	1241 (44.3)	292 (33.0)	73 (34.1)	43 (45.7)	1649		
Large-scale purchases of masks							
Yes	1433 (51.2)	564 (63.7)	128 (59.8)	56 (59.6)	2181	45.971	<0.001
No	1368 (48.8)	322 (36.3)	86 (40.2)	38 (40.4)	1814		
Unreasonable purchases of oxygen generators							
Yes	73 (2.6)	21 (2.4)	9 (4.2)	5 (5.3)	108	4.756^a^	0.164^a^
No	2728 (97.4)	865 (97.6)	205 (95.8)	89 (94.7)	3887		

^a^Fisher’s exact test

### Influential factors of coping behavior among participants

**Fig. 5:**
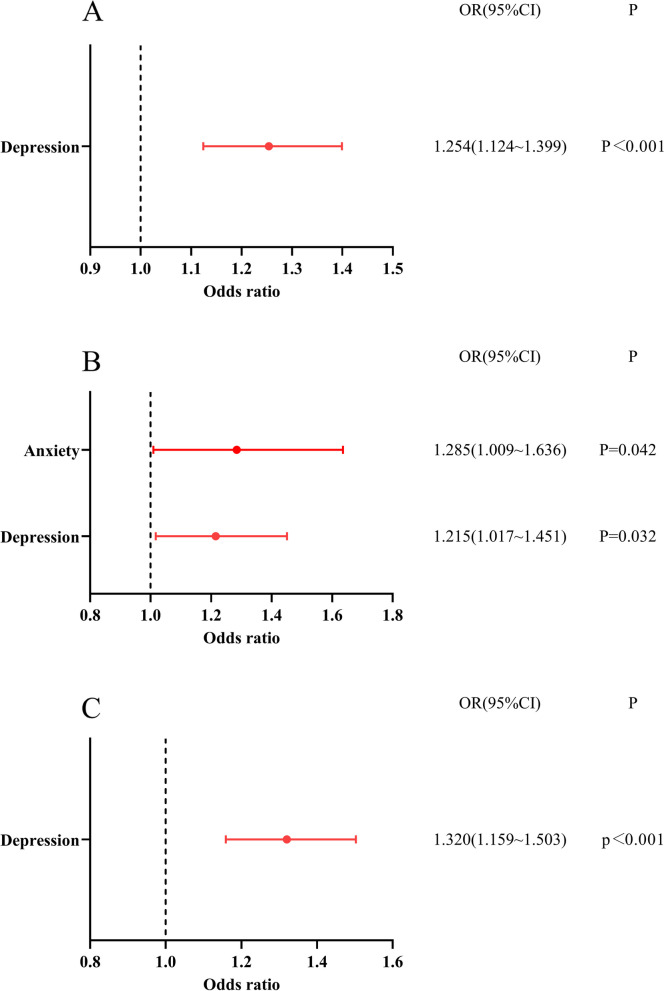
Logistic regression of medical treatment: **A** shows logistic regression of self-medication. **B** shows logistic regression of medical institution visits. **C** shows logistic regression of online medical services

The binary logistic regression results of self-medication and clinical treatment behavior showed that depression was a risk factor affecting self- medication (OR = 1.254, 95%CI: 1.124 ~ 1.399), seeking professional treatment (OR = 1.215, 95%CI: 1.017 ~ 1.451) and using online services of medical institutions (OR = 1.320, 95%CI: 1.159 ~ 1.503) (Fig. 5A, B, and C); anxiety was a risk factor affecting seeking professional treatment (OR = 1.285, 95%CI: 1.009 ~ 1.636) (Fig. 5B).

**Fig. 6:**
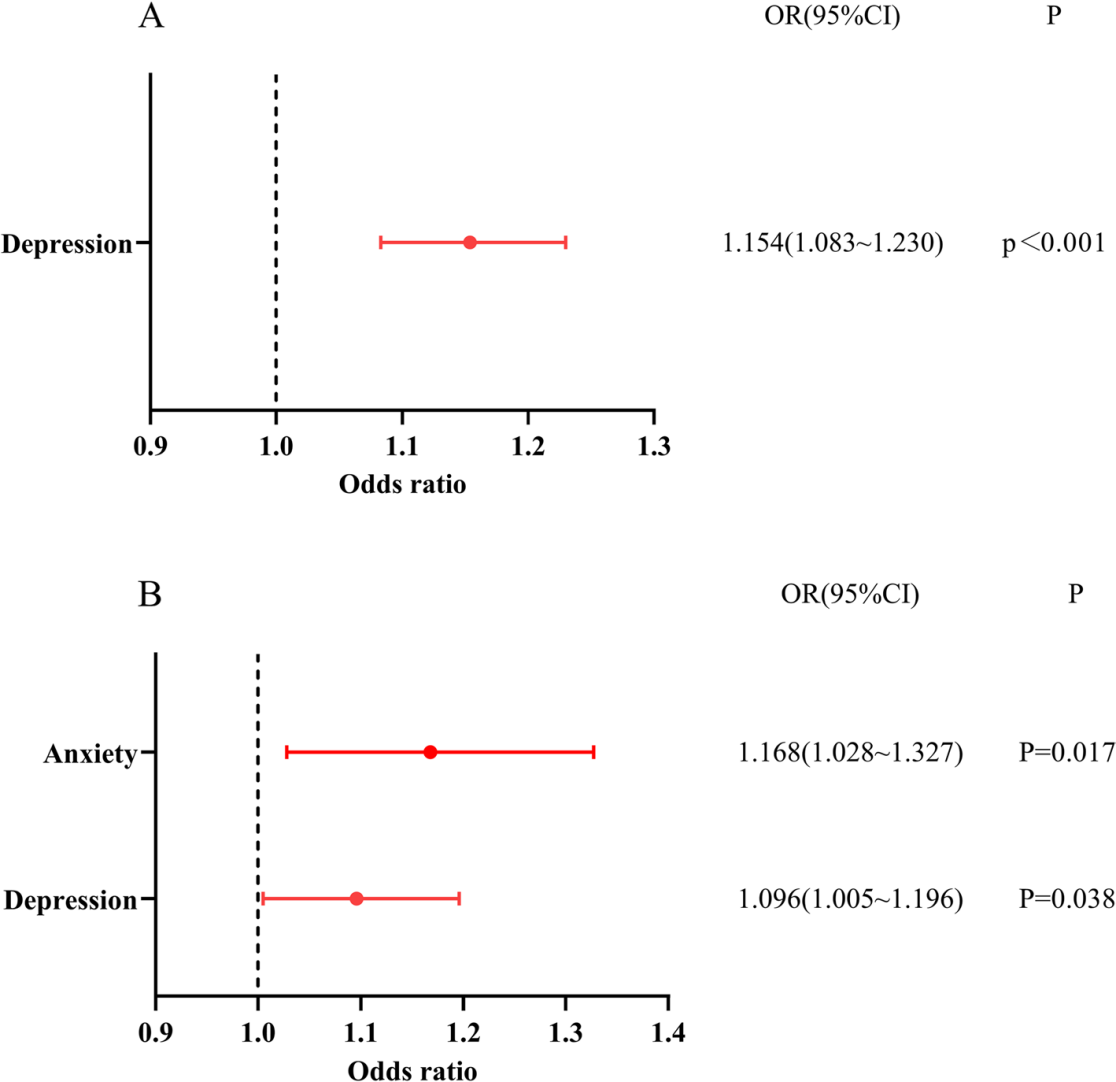
Logistic regression of irrational purchase behavior: **A** shows logistic regression of buying medicines in large quantities. **B** shows logistic regression of buying masks in large quantities

The binary logistic regression results of irrational purchase behavior showed that depression was a risk factor affecting large-scale purchases of medicines (OR = 1.154, 95%CI: 1.083 ~ 1.230) and masks (OR = 1.096, 95%CI: 1.005 ~ 1.196) (Fig. 6A, and B); anxiety was a risk factor affecting large-scale purchases of masks (OR = 1.168, 95%CI: 1.028 ~ 1.327) (Fig. 6B).

## Discussion

This study evaluates the changes in the negative emotions of the population after the adjustment of COVID-19 epidemic prevention and control policy and analyzes the impact of depression and anxiety on medical treatment and irrational purchase behavior of people infected with COVID-19. The infection of COVID-19 had a negative impact on people’s mental health, the incidences of depression and anxiety were 59.1% and 29.9%. At the same time, we found that negative emotions are the influencing factors of people’s self- medication, seeking professional treatment, using online services of medical institutions, large-scale purchases of medicines and masks. We must pay more attention to people’s mental health and respond to coping behaviors caused by negative emotions.

Clinical treatment Affected by depression, anxiety, after the adjustment of COVID-19 epidemic prevention and control policy. Depression and anxiety are risk factors for self-medication and medical behavior. When the level of depression or anxiety increases, the possibility of self- medication, seeking professional treatment, and using online services of medical institutions will increases. A survey from India [14] showed that 24.2% of the public had taken medicine without a prescription, and more self-medication appeared among anxious respondents. Self-medication occurred in most patients (88.9%) in our study and more frequently in patients with depression (90.8%, None is 86.1%) and anxiety (91.2%, None is 87.9%). Although it is more convenient and quick for patients to relieve disease symptoms and reduce economic burden by self-medication [15], but the risks of self-medication are also worthy of attention. Self-medication may lead to polypharmacy, incorrect diagnosis, adverse effects, drug interactions, antibiotic resistance, and increased drug expenses [16–18]. Research by M. Gras et al. [19] pointed out that the proportion of reported adverse drug reactions linked to self-medication was higher during the COVID-19 outbreak, and medication errors and overdoses accounted for 31.5% and 31% of adverse drug reactions linked to self-medication, respectively. Therefore, more attention should be paid to medication guidance for the public to improve public awareness of safe and rational use of medicines.

After the adjustment of COVID-19 epidemic prevention and control policy, the level of depression or anxiety is a key factor affecting irrational consumption behavior, and when the level increases, people are more likely to consume irrationally. A study [20] showed that risk perception, state anxiety, and trust in social media trigger irrational consumption behavior, with state anxiety being the most important factor to induce such behavior. Another study on anxiety in India [21] pointed out that Media reporting about the shortage of resources and essential things of daily living further increases the panic buying. Similar to the findings of Lins, S. et al. [22], we believe that under the background of policy adjustment from strict control to flexible control, the COVID-19 pandemic may still induce negative emotions among individuals, raising concerns about the adequacy of supplies of medicines or masks. At the same time, the excessive buying and storage behaviors can cause demands that overcome the supply, generating product shortages in the middle of the crisis, or even triggering a herd panic buying, increasing feelings of instability and anxiety.

### Strength and limitations

This study focuses on the psychological impact of large adjustments in government policies on people in the face of highly contagious viruses. Provide reference and suggestions for government policy formulation. At the same time, under the background of policy adjustment from strict control to flexible control, the research on the change of people’s psychology and corresponding coping behavior is still insufficient.

The limitations of this study still require our attention. During the outbreak of COVID-19, due to the shortage of medical resources, some participants were unable to perform antigen or nucleic acid detection in time, so their reported infection status was infected or highly suspected infected, which may affect the representativeness of the sample for COVID-19 patients. In addition, most of the participants were people with higher education or living in cities, which shows that we cannot adequately reflect the psychology and coping behaviors of groups such as rural areas or low-education groups, so it is suggested that future studies should include more participants from rural areas or low-education groups.

## Conclusion

After the adjustment of COVID-19 epidemic prevention and control policy, patient risk perception can increase depression and anxiety. We found that associated with depression, COVID-19 patients are more likely to have medical behaviors such as self- medication, seeking professional treatment, using online services of medical institutions, and storage behaviors of medicines and masks; and anxiety associated with the coping behavior of patients to seek professional treatment and store masks in large quantities. On the one hand, we should improve people’s mental health, and on the other hand, we should give people effective psychological education during the epidemic. Therefore, we should set up psychological outpatient clinics in community health institutions, expanding mental health screening and guidance; relying on the psychological outpatient clinic, establish groups of people with depression or anxiety to carry out COVID-19 health education and peer education, to reduce adverse drug reactions, avoid panic seeking professional treatment and irrational purchase behavior, and protect public mental health. It is worth noting that the causal relationship between infection and psychology still needs to be confirmed by follow-up studies.

## Data Availability

The datasets generated and/or analyzed during the current study are not publicly available due to database containing participants’ personal information but are available from the corresponding author on reasonable request.

## Abbreviations

PHQ-9: The Patient Health Questionnaire
GAD-7: The General Anxiety Disorder
WHO: The World Health Organization
SM: Self-medication
SD: Standard deviation
OR: Odds ratios
95% CI: 95% confidence intervals
